# TssA from *Aeromonas hydrophila*: expression, purification and crystallographic studies

**DOI:** 10.1107/S2053230X18010439

**Published:** 2018-09-03

**Authors:** Samuel R. Dix, Ruyue Sun, Matthew J. Harris, Sarah L. Batters, Svetlana E. Sedelnikova, Patrick J. Baker, Mark S. Thomas, David W. Rice

**Affiliations:** a Department of Molecular Biology and Biotechnology, University of Sheffield, Firth Court, Western Bank, Sheffield S10 2TN, England; b Department of Infection, Immunity and Cardiovascular Disease, University of Sheffield Medical School, Beech Hill Road, Sheffield S10 2RX, England; c Department of Chemistry, King’s College London, Britannia House, London SE1 1DB, England

**Keywords:** type VI secretion system, TssA subunit, *Aeromonas hydrophila*

## Abstract

TssA is a core subunit of the type VI secretion system, which is a major player in interspecies competition in Gram-negative bacteria. Previous studies on enteroaggregative *Escherichia coli* TssA suggested that it is comprised of three putative domains: a conserved N-terminal domain, a middle domain and a ring-forming C-terminal domain. X-ray studies of the latter two domains have identified their respective structures. Here, the results of the expression and purification of full-length and domain constructs of TssA from *Aeromonas hydrophila* are reported, resulting in diffraction-quality crystals for the middle domain (Nt2) and a construct including the middle and C-terminal domains (Nt2-CTD).

## Abstract 2

Crystals of different domain constructs of the TssA subunit from *Aeromonas hydrophila* have been crystallized in forms suitable for X-ray analysis.

## 1. Introduction   

The type VI secretion system (T6SS) is a multiprotein complex found in Gram-negative bacteria that is involved in the delivery of various effector proteins into target cells. It is thought to be evolutionarily related to the contractile tail assembly of bacteriophages (for example T4) and to R-type pyocins, which all share a similar contraction mechanism (Leiman *et al.*, 2009 ▸; Basler *et al.*, 2012 ▸; Ge *et al.*, 2015 ▸). These nanomachines utilize a platform known as the baseplate, onto which a tube, surrounded by a contractile sheath, is polymerized. The inner tube is capped at the baseplate end with proteins that provide a tapered end, which facilitates the penetration of target cells upon contraction of the sheath against the baseplate (Leiman *et al.*, 2009 ▸; Shneider *et al.*, 2013 ▸; Ge *et al.*, 2015 ▸). The T6SS is comprised of multiple copies of 13 core subunits, and a single PAAR tip protein, that collectively can be categorized into two major components. The first is the membrane complex, consisting of ten copies each of TssJ, TssL and TssM. Together, these form a transmembrane assembly which acts as an ‘anchor’ for the other component, the ‘injection machinery’. In addition, it allows the passage of the contractile tube and the associated effector proteins out of the bacterium (Zheng & Leung, 2007 ▸; Boyer *et al.*, 2009 ▸; Durand *et al.*, 2015 ▸; Cianfanelli *et al.*, 2016 ▸). The injection machinery consists of two subcomplexes. One of these contains the tube, which is comprised of stacked hexamers of TssD (Hcp) and is capped by a trimer of TssI (VgrG) and a single PAAR subunit. The tube is surrounded by polymerized heterodimers of TssBC, arranged in a six-start helix, that form the contractile sheath (Basler *et al.*, 2012 ▸; Shneider *et al.*, 2013 ▸; Brunet *et al.*, 2014 ▸; Kube *et al.*, 2014 ▸; Wang *et al.*, 2017 ▸). The tube and sheath are assembled on the second subcomplex, the baseplate, which contains TssE, TssF, TssG and TssK. During contraction of the sheath against the baseplate, the inner tube and associated effector proteins are driven through an opening in the centre of the baseplate (Pukatzki *et al.*, 2007 ▸; Brunet *et al.*, 2015 ▸; Taylor *et al.*, 2016 ▸). Post-construction, the sheath is then recycled by the AAA+ ATPase TssH (ClpV; Bönemann *et al.*, 2009 ▸; Basler *et al.*, 2012 ▸).

Until recently, very little was known regarding the role and location of the TssA subunit within the T6SS complex. It has been shown that all TssAs comprise a conserved N-terminal region containing a domain of unknown function termed ImpA_N (Pfam PF0681224; Finn *et al.*, 2016 ▸) in addition to a divergent C-terminal region (Shalom *et al.*, 2007 ▸; Zoued *et al.*, 2017 ▸). Recent fluorescence microscopy and cryo-EM structural studies on enteroaggregative *Escherichia coli* (EAEC) and *Vibrio cholerae* T6SS, respectively, have shown that TssA is located and maintained at the baseplate distal end of the growing TssBCD complex during tube and sheath polymerization (Zoued *et al.*, 2016 ▸; Nazarov *et al.*, 2018 ▸). Furthermore, proteolytic and structural studies of domains from EAEC TssA (Ec042_4540; Ec-TssA) have identified a putative three-domain organization corresponding to an N-terminal domain, a middle domain and a C-terminal domain, and have provided X-ray structures for the latter two. This revealed that the C-terminal domain is organized into a six-pointed star consisting of 12 subunits with *D*_6_ symmetry (Zoued *et al.*, 2016 ▸).

We have observed that some TssA homologues are ∼40 amino acids shorter at the C-terminus compared with Ec-TssA. This prompted an investigation into one such TssA subunit, AHA1844, that is present in *Aeromonas hydrophila* ATCC 7966. In this paper, we report the generation of suitable constructs for the crystallization of *A. hydrophila* TssA (Ah-TssA) and its constituent domains (N-terminal domain, Nt1; middle domain, Nt2; C-terminal domain, CTD). Furthermore, we present the first crystallization trials and X-ray diffraction data for proteins derived from constructs of both the Ah-TssA Nt2 and Nt2-CTD domains as initial steps to determining the structures of these domains and their relationship to EAEC TssA.

## 2. Materials and methods   

### 2.1. Macromolecule production   

**Figure 1:**
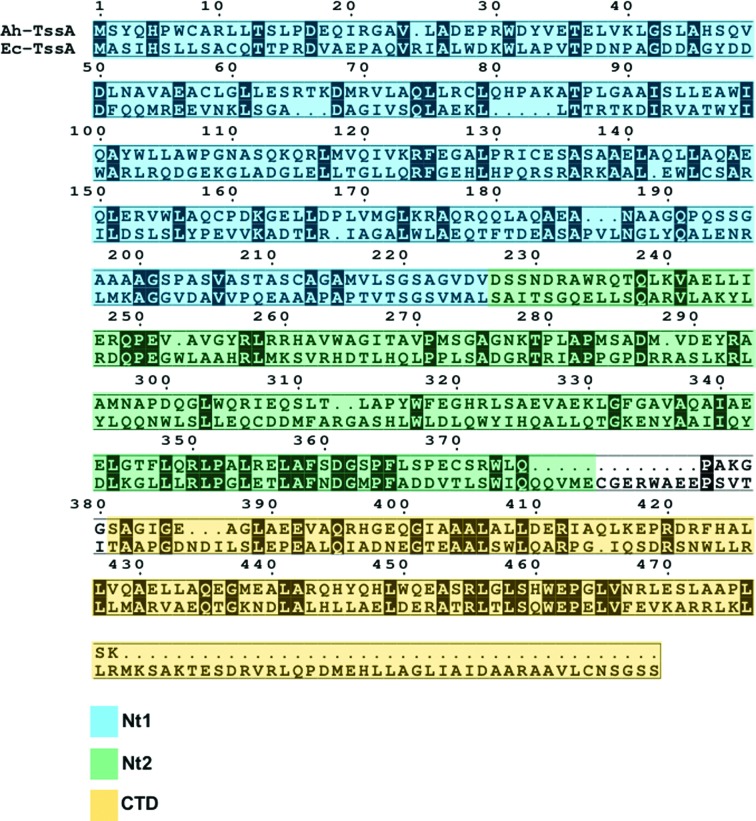
Putative domain boundaries of Ah-TssA and Ec-TssA. Proteolytic and structural studies of Ec-TssA identified putative domain boundaries for Nt1, Nt2 and CTD (Zoued *et al.*, 2016 ▸). This information was used along with a sequence alignment between Ah-TssA and Ec-TssA to design suitable Ah-TssA domain constructs for crystallization. The key indicates the colour assigned to each putative domain.

Domain boundaries for Nt1, Nt2 and CTD were defined using an amino-acid sequence alignment of Ec-TssA and Ah-TssA (Fig. 1 ▸). DNAs encoding Ah-TssA or Ah-TssA domains (UniProt A0KJC7) were amplified from *A. hydrophila* ATCC 7966 (Seshadri *et al.*, 2006 ▸) by the polymerase chain reaction (PCR) utilizing the primer pairs described in Table 1 ▸. The products were then ligated into the pACYCDuet-1 expression vector (Novagen) downstream of the first T7 promoter using compatible restriction sites. The recombinant expression plasmids containing *tssa* DNA were transformed into *E. coli* strain JM83 (Yanisch-Perron *et al.*, 1985 ▸), verified by nucleotide sequencing and then transformed into *E. coli* BL21(DE3) cells (Novagen; Studier & Moffatt, 1986 ▸) for protein overproduction (Hanahan, 1983 ▸).

Ah-TssA constructs representing the full-length protein, the three identified domains (Nt1, Nt2 and CTD) and a combination of two of them (Nt2-CTD) were grown in 2 l BHI broth (Oxoid) at 37°C with 25 µg ml^−1^ chloramphenicol. Once the cultures had reached an OD_600_ of 0.5–0.7, IPTG was added to a final concentration of 1 m*M* (0.1 m*M* for His6.Ah-TssA Nt2) for the induction of the T7 promoter present within each expression vector (Table 1 ▸). Cultures were incubated for a further 2–3 h at 37°C (30°C was used to induce the synthesis of His6.Ah-TssA, His6.Ah-TssA Nt1 and His6.Ah-TssA Nt2) before the cells were harvested. The harvested cells were resuspended in 50 m*M* Tris–HCl pH 8.0 containing 2 m*M* EDTA, 200 m*M* NaCl, 10% glycerol (5 ml per gram of cell paste), treated with lysozyme (0.2 mg ml^−1^) for 30 min at 4°C followed by sodium deoxycholate (0.5 mg ml^−1^) and PMSF (25 µg ml^−1^) for a further 30 min, and lysed by sonication [EDTA was omitted and imidazole (10 m*M* final concentration) was included if the protein was to be purified by nickel-affinity chromatography, and the lysozyme and sodium deoxy­cholate steps were omitted if the cell paste was frozen before the sonication step]. The lysate was then cleared by centrifugation for 30 min at 35 000*g*.

His-tagged proteins (*i.e.* all constructs except Ah-TssA CTD) were applied onto a 5 ml HisTrap HP 5 ml column (GE Healthcare Life Sciences) and eluted with a linear gradient of imidazole (10–500 m*M*). His6.Ah-TssA was further purified by SEC (Superose 6 10/300 GL, GE Healthcare Life Sciences) in 50 m*M* Tris–HCl pH 8.0 containing 500 m*M* NaCl. The Ah-TssA CTD lysate was first precipitated with 4 *M* ammonium sulfate and the pellet was subsequently resuspended in 50 m*M* Tris–HCl pH 8.0 and applied onto an anion-exchange column (Resource Q 6 ml, GE Healthcare Life Sciences) before being eluted with a linear gradient of NaCl (0–500 m*M*). Ah-TssA CTD was further purified by SEC (HiLoad Superdex 200 16/600, GE Healthcare Life Sciences) in 50 m*M* Tris–HCl pH 8.0 containing 500 m*M* NaCl. Protein purity was analysed by SDS–PAGE and subsequent staining.

### 2.2. Crystallization   

Crystallization trials were performed on all purified Ah-TssA constructs using the protein concentration and protein buffer compositions shown in Table 2 ▸. Initial screening was carried out using a Matrix Hydra II Plus One crystallization robot dispensing into 96-well MRC2 sitting-drop crystallization trays, whereby a 1:1 ratio of precipitant:protein was created generating 400 nl drops, which were allowed to equilibrate through vapour diffusion at 290 K (280 K for Ah-TssA Nt2-CTD). Commercially available sparse-matrix screens (pH Clear, Morpheus, PACT, JCSG+, MPD and ProPlex) were used to identify conditions that formed crystals. Successful crystallization conditions for each construct, if applicable, can be found in Table 2 ▸. Optimization of conditions was carried out when required in 24-well trays with a reservoir volume of 500 µl and a drop size of 2 µl. A mercury derivative of the His6.Ah-TssA Nt2 crystals was generated by the addition of powdered ethylmercury phosphate (EMP; ∼20 µg) to a drop containing crystals to provide a derivative.

### 2.3. Data collection and processing   

Crystals were harvested in cryoloops (Hampton Research) and immediately soaked in a solution consisting of mother liquor with an additional 25%(*v*/*v*) ethylene glycol [supplemented with an additional 10%(*v*/*v*) glycerol for crystals of His6.Ah-TssA Nt2] for about 10 s before being flash-cooled to 100 K in liquid nitrogen. Native data were collected for crystals of His6.Ah-TssA Nt2 and His6.Ah-TssA CTD-Nt2 on MX beamlines I03 and I04 at Diamond Light Source, Oxford, respectively. In addition, data for a mercury derivative of crystals of His6.Ah-TssA Nt2 were collected on beamline I03. Diffraction data were collected as shown in Table 3 ▸ and the data were processed utilizing the *xia*2 software pipeline (Winter, 2010 ▸; Winter & McAuley, 2011 ▸) with/without running the optional *XDS* package (Kabsch, 2010 ▸) as indicated. All details of data processing are shown in Table 3 ▸. The estimated content of the asymmetric unit and the self-rotation function were determined using the *CCP*4 suite (Winn *et al.*, 2011 ▸).

## 3. Results and discussion   

The full-length TssA construct, His6.Ah-TssA, and the four different domain constructs were all successfully overproduced and purified as described. The final protein yields after purification were 14 mg for His6.Ah-TssA, 5.6 mg for His6.Ah-TssA Nt1, 2.7 mg for His6.Ah-TssA Nt2, 7.2 mg for Ah-TssA CTD and 16 mg for His6Ah-TssA Nt2-CTD. Analysis by gel filtration indicates that His6.Ah-TssA Nt1 is monomeric and that His6.Ah-TssA Nt2 is present as a dimer, whereas gel filtration of His6.Ah-TssA, Ah-TssA CTD and His6.Ah-TssA Nt2-CTD indicated approximate molecular weights of 0.6, 0.2 and 0.4 MDa, respectively, indicating the presence of larger complexes.

**Figure 2:**
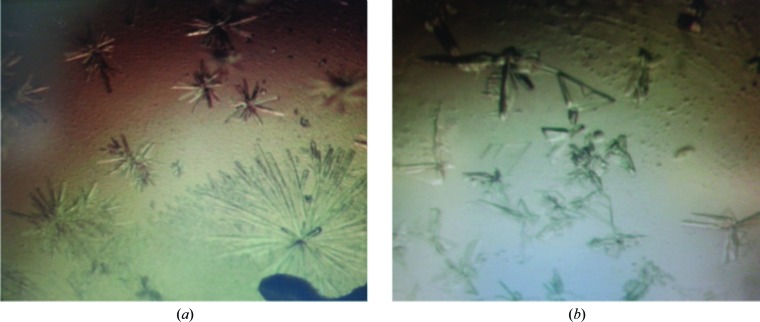
Crystals of Ah-TssA domains. (*a*) His6.Ah-TssA Nt2 crystals grown in 0.04 *M* potassium phosphate monobasic, 16%(*w*/*v*) PEG 8000, 20%(*v*/*v*) glycerol. (*b*) His6.Ah-TssA Nt2-CTD crystals grown optimally in 0.2 *M* sodium acetate, 0.1 *M* sodium citrate pH 5.5, 10%(*w*/*v*) PEG 4000.

The full-length protein and the four domain constructs were subjected to crystallization trials, and two (His6.Ah-TssA Nt2 and His6.Ah-TssA Nt2-CTD) produced crystals suitable for data collection at Diamond Light Source, Oxford (Fig. 2 ▸). SDS–PAGE analysis of the two purified protein constructs (His6.Ah-TssA Nt2 and His6.Ah-TssA Nt2-CTD) indicated initial purities of approximately >95% (Supplementary Fig. S1). The crystals diffracted to 2.05 and 3.75 Å resolution, respectively. The asymmetric unit of the His6.Ah-TssA Nt2 crystals is estimated to contain two molecules, with a probable Matthews coefficient of 2.53 Å^3^ Da^−1^. Calculating the probable Matthews coefficient for the data from the His6.Ah-TssA Nt2-CTD crystals suggested an asymmetric unit containing between 10 and 18 subunits. Analysis of a self-rotation function based on all data for 4–16 Å resolution with a range of integration radii gave no indication as to the symmetry of His6.Ah-TssA Nt2-CTD within the asymmetric unit. Preliminary data to 2.3 Å resolution from the mercury-soaked His6.Ah-TssA Nt2 crystals provided an initial map to which the sequence could be fitted, indicating that the crystals were of the Nt2 domain (Supplementary Fig. S2 ▸). Refinement of the structure and attempts to collect data to higher resolution are currently ongoing.

## Supplementary Material

Supplementary Figures.. DOI: 10.1107/S2053230X18010439/rl5161sup1.pdf

## Figures and Tables

**Table 1:** Primers and construct design Bold sequences indicate restriction sites.

Construct	His6.Ah-TssA	His6.Ah-TssA Nt1	His6.Ah-TssA Nt2	Ah-TssA CTD	His6.Ah-TssA Nt2-CTD
Forward primer	GCGC**AGATCT**AATGAGCTATCAACACCCCTG	GCGC**AGATCT**AATGAGCTATCAACACCCCTG	GCGC**GGATCC**AGGCGTCGACGTCGACAGTTC	GCGC**GGATCC**GAGTGCGGGCATTGGCGAGGC	GCGC**GGATCC**AGGCGTCGACGTCGACAGTTC
Reverse primer	GCGC**AAGCTT**TCATTTCGACAACGGCGCCG	GCGC**GGTACC**TTAGCTGGAACTGTCGACGTCGACG	GCGC**AAGCTT**TTAAGCCTCGCCAATGCCCGCACT	GCGC**AAGCTT**TCATTTCGACAACGGCGCCG	GCGC**AAGCTT**TCATTTCGACAACGGCGCCG
Cloning/expression vector	pACYCDuet-1	pACYCDuet-1	pACYCDuet-1	pACYCDuet-1	pACYCDuet-1
Restriction sites	BamHI and HindIII	BamHI and KpnI	BamHI and HindIII	BamHI and HindIII	BamHI and HindIII
Expression host	*E. coli* BL21(DE3)	*E. coli* BL21(DE3)	*E. coli* BL21(DE3)	*E. coli* BL21(DE3)	*E. coli* BL21(DE3)
Amino acids	His_6_.(1–478) (52.6 kDa)	His_6_.(1–229) (26.0 kDa)	His_6_.(223–387) (19.7 kDa)	381–478 (10.7 kDa)	His_6_.(223–478) (29.8 kDa)

**Table 2:** Crystallization N/A: constructs that did not crystallize.

Construct	His6.Ah-TssA	His6.Ah-TssA Nt1	His6.Ah-TssA Nt2	Ah-TssA CTD	His6.Ah-TssA Nt2-CTD
Method	Vapour diffusion	Vapour diffusion	Vapour diffusion	Vapour diffusion	Vapour diffusion
Plate type	96-well MRC2	96-well MRC2	24-well optimization tray	96-well MRC2	24-well optimization tray
Temperature (K)	290	290	290	290	280
Protein concentration (mg ml^−1^)	10	10	10	18	11
Buffer composition of protein solution	50 m*M* Tris–HCl pH 7.5, 100 m*M* NaCl	50 m*M* Tris–HCl pH 8.0, 50 m*M* NaCl	10 m*M* Tris–HCl pH 8.0	10 m*M* Tris–HCl pH 8.0	10 m*M* Tris–HCl pH 8.0
Composition of reservoir solution	N/A	N/A	0.04 *M* potassium phosphate monobasic, 16%(*w*/*v*) PEG 8000, 20%(*v*/*v*) glycerol	N/A	0.2 *M* sodium acetate, 0.1 *M* sodium citrate pH 5.5, 10%(*w*/*v*) PEG 4000

**Table 3:** Data collection and processing Values in parentheses are for the outer shell.

Construct	His6.Ah-TssA Nt2	His6.Ah-TssA Nt2	His6.Ah-TssA Nt2-CTD
Crystal	Native	EMP derivative	Native
Diffraction source	I03	I03	I04
Wavelength (Å)	0.9763	1.0088	0.9795
Temperature (K)	100	100	100
Detector	PILATUS 6M-F	PILATUS 6M-F	PILATUS 6M-F
Rotation range per image (°)	0.1	0.1	0.1
Total rotation range (°)	200	360	200
Exposure time per image (s)	0.05	0.05	0.1
Data-processing package	*xia*2 -3dii	*xia*2 -3dii	*DIALS*
Space group	*P*2_1_	*P*2_1_	*P*2_1_
*a*, *b*, *c* (Å)	46.0, 40.1, 101.0	46.1, 40.1, 100.8	73.0, 202.7, 137.7
α, β, γ (°)	90.0, 103.2, 90.0	90, 102.7, 90	90.0, 92.3, 90.0
Cell volume (Å^3^)	181631	182001	2036168
Resolution range (Å)	2.05 (2.10–2.05)	2.28 (2.34–2.28)	3.75 (3.85–3.75)
*R*_meas_	0.142 (1.315)	0.176 (1.488)	0.117 (0.950)
CC_1/2_	0.995 (0.574)	0.996 (0.532)	0.862 (0.757)
〈*I*/σ(*I*)〉	6.2 (1.2)	7.3 (1.0)	6.8 (1.5)
Completeness (%)	98.8 (99.2)	99.3 (95.1)	100.0 (100.0)
Multiplicity	3.6 (3.7)	6.4 (5.0)	3.7 (3.7)
Total reflections	81939 (6224)	107275 (5874)	153004 (11196)
Unique reflections	22646 (1700)	16664 (1183)	41019 (3066)
Overall *B* factor from Wilson plot (Å^2^)	31	42	170
Anomalous completeness (%)	—	98.9 (93.2)	—
Anomalous multiplicity	—	3.3 (2.6)	—
Anomalous correlation	—	0.217 (−0.022)	—
Anomalous slope	—	1.105	—
