# Supplementary material for: TssA from Aeromonas hydrophila: expression, purification and crystallographic studies
Source: Acta Crystallogr F Struct Biol Commun. 2018 Sep 3;74(Pt 9):578–82. doi: 10.1107/S2053230X18010439 (PMC6130423; doi:10.1107/S2053230X18010439)
Supplement: Supplementary file 1 [file f-74-00578-sup1.pdf]

Acta Cryst F

STRUCTURAL BIOLOGY COMMUNICATIONS

Volume 74 (2018)

Supporting information for article:

TssA from *Aeromonas hydrophila*: expression, purification and crystallographic studies

Samuel R. Dix, Ruyue Sun, Matthew J. Harris, Sarah L. Batters, Svetlana E. Sedelnikova, Patrick J. Baker, Mark S. Thomas and David W. Rice

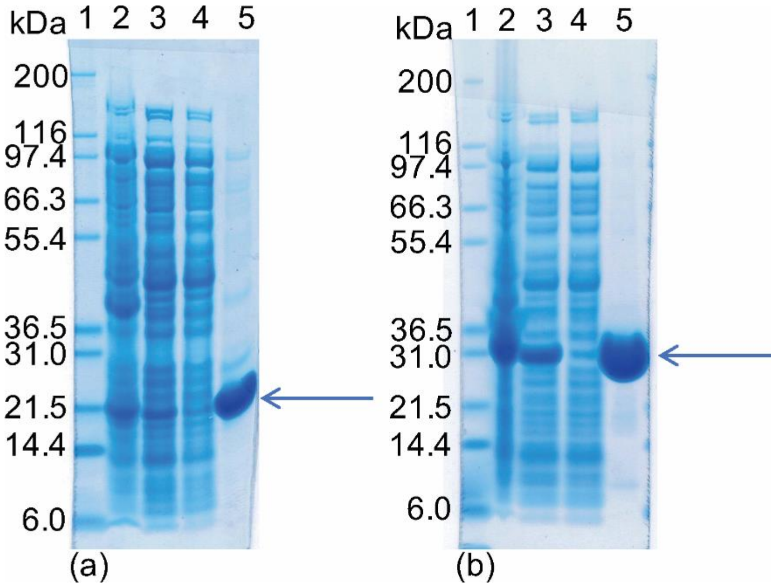

Figure S1 SDS-PAGE analysis of protein purification for *A. hydrophila* TssA His_6.Ah-TssA Nt2 and His_6.Ah-TssA Nt2-CTD constructs. (*a*) Purification analysis of the His_6.Ah-TssA Nt2 construct. (*b*) Purification analysis of the His_6.Ah-TssA Nt2-CTD construct. Lane 1 - Mark12 ladder, Lane 2 - Cell debris, Lane 3 - Cell free extract, Lane 4 - Unbound fraction, Lane 5 - Final preparation of construct. Gels indicate successful purification of their respective constructs, protein of interest indicated by arrow, protein appears >95 % purity in the final preparation lanes.

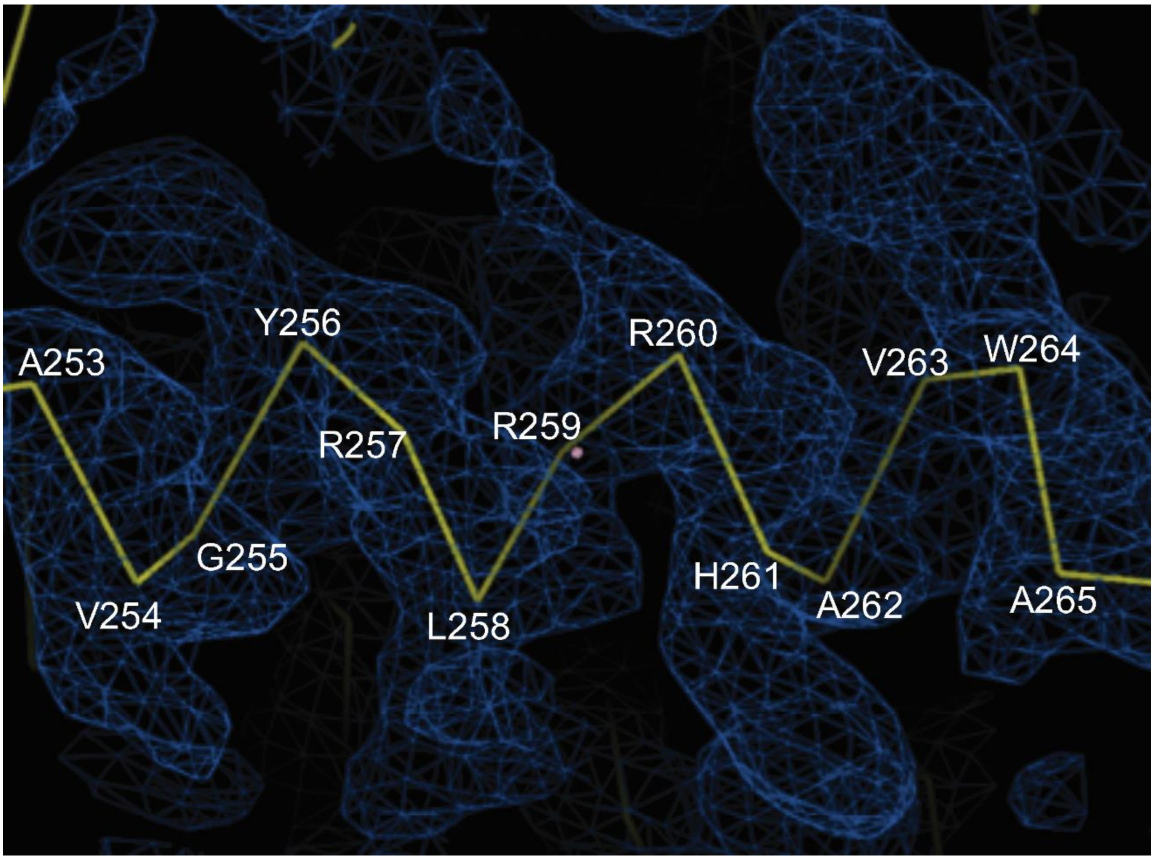

Figure S2 Preliminary chain trace through a helical region of the Nt2 domain of *A. Hydrophila* TssA, between residues A253 - A265. Large side chains corresponding to Y256, R260, H261 and W264 can be identified.
